# Effect of a Six-Month Lifestyle Intervention on the Physical Activity and Fitness Status of Adults with NAFLD and Metabolic Syndrome

**DOI:** 10.3390/nu14091813

**Published:** 2022-04-26

**Authors:** Catalina M. Mascaró, Cristina Bouzas, Sofia Montemayor, Miguel Casares, Isabel Llompart, Lucía Ugarriza, Pere-Antoni Borràs, J. Alfredo Martínez, Josep A. Tur

**Affiliations:** 1 Research Group on Community Nutrition and Oxidative Stress, University of the Balearic Islands-IUNICS, 07122 Palma de Mallorca, Spain; c.mascaro@uib.es (C.M.M.); cristina.bouzas@uib.es (C.B.); sofiamf16@gmail.com (S.M.); isabel.llompart@ssib.es (I.L.); luciaugarriza@gmail.com (L.U.); 2 Health Institute of the Balearic Islands (IDISBA), 07120 Palma de Mallorca, Spain; 3 CIBEROBN (Physiopathology of Obesity and Nutrition CB12/03/30038), Instituto de Salud Carlos III (ISCIII), 28029 Madrid, Spain; 4 Radiodiagnosis Service, Red Asistencial Juaneda, 07011 Palma de Mallorca, Spain; casaresmiguel@gmail.com; 5 Clinical Analysis Service, University Hospital Son Espases, 07120 Palma de Mallorca, Spain; 6 Camp Redó Primary Health Care Center, 07010 Palma de Mallorca, Spain; 7 Area of Physical Education and Sports, Department of Pedagogy and Specific Didactics, University of the Balearic Islands, 07122 Palma de Mallorca, Spain; pa-borras@uib.es; 8 Cardiometabolics Precision Nutrition Program, IMDEA Food, CEI UAM-CSIC, 28049 Madrid, Spain; jalfredo.martinez@imdea.org; 9 Department of Nutrition, Food Sciences, and Physiology, University of Navarra, 31008 Pamplona, Spain

**Keywords:** physical activity, Mediterranean diet, non-alcoholic fatty liver disease, metabolic syndrome, fitness

## Abstract

(1) Background: Physical inactivity has been linked to NAFLD, and exercise has been reported as useful to reduce intrahepatic fat content in NAFLD. (2) Objectives: To assess the physical activity (PA) and fitness status after a six-month lifestyle intervention (diet and PA) in adults with NAFLD and metabolic syndrome (MetS). (3) Design: Prospective cohort analysis of data obtained between baseline and six-year parallel-group randomized trial (n = 155, aged 40–60 years old, with MetS and NAFLD). Participants were randomized into three nutritional and PA intervention groups: Conventional diet (CD); MedDiet-high meal frequency (MD-HMF); MedDiet-physical activity (MD-PA). (4) Methods: PA and fitness status were assessed using a validated Minnesota questionnaire, ALPHA-FIT test battery, accelerometers, and functional fitness score. Information related to age, gender, education level, marital status, socioeconomic status, smoking habit, and alcohol consumption were also obtained. (5) Results: The CD group had higher improvement in standing handgrip than the MD-HMF group. The MD-PA group did more modified push-up repetitions than the CD group. The MD-PA and CD groups showed higher sitting handgrip than the MD-HMF group. The MD-HMF group showed the highest decrease in aerobic capacity. The MD-PA group showed lower light intensity PA/day than the CD and MD-HMF groups. The MD-PA group showed higher moderate intensity PA than the CD and MD-HMF groups. The CD group reported more METs per day than the MD-HMF group. (6) Conclusions: Lifestyle six-month intervention with diet and regular PA improved functional fitness in middle-aged patients with NAFLD and MetS. Aerobic capacity improved in patients who followed a Mediterranean diet and regular training sessions at six months.

## 1. Introduction

A current common cause of liver disease in the western world is non-alcoholic fatty liver disease (NAFLD), which is defined as the excess deposition of fat in the hepatocytes [1]. It can evolve into inflammatory steatohepatitis (NASH), fibrosis, cirrhosis and hepatocellular carcinoma [2,3]. NAFLD is not induced by an excess of alcohol or drugs consumption [4], and is considered to be the manifestation of metabolic syndrome (MetS) [5,6]. Visceral obesity and insulin resistance/type 2 diabetes mellitus are tightly related to NAFLD, but also dyslipidemia and hypertension are potential risk factors for the disease [7,8,9]. Modifying lifestyle to improve and prevent metabolic risk factors is the existent treatment for NAFLD and NASH. A Mediterranean diet and physical activity (PA) are proposed as primary therapy to reduce liver fat and reverse NAFLD [10,11].

A Mediterranean diet can improve, or prevent, MetS, type 2 diabetes mellitus or cardiovascular diseases because it is a diet rich in antioxidants, unsaturated fatty acids and fiber, while being low in animal proteins and saturated fats [12]. In this way, the Mediterranean diet improves lipid profile and insulin resistance, thereby also preventing NAFLD-related diseases [13].

Physical inactivity has been linked to NAFLD; people with NAFLD are more sedentary and/or sit for a prolonged time [14]. It has been shown that, like a Mediterranean diet, regular PA improves components related to MetS and type 2 diabetes mellitus. An active lifestyle controls low levels of plasma high density lipoprotein cholesterol (HDL-c) and reduces weight, hypertriglyceridemia and hypertension [4].

It was reported that exercise is useful to reduce intrahepatic fat content in subjects with NAFLD, even without weight loss, but this effect is greater when subjects lose weight [15]. A combination of PA and diet resulted in higher weight loss than diet alone [16], but also in higher improvement in risk factors for NAFLD than PA or diet alone [17]. However, evidence on the combined effect of diet and PA on NAFLD is still scarce.

The aim of the current study was to assess PA and fitness status after a six-month lifestyle intervention (diet and PA) in adults with NAFLD and MetS.

## 2. Methods

### 2.1. Design

The current trial was a prospective cohort analysis of data obtained between baseline and a 6-year parallel-group randomized trial. The aim of the trial was to assess the effect of a 6-month dietary and physical activity intervention on NAFLD status in adults with MetS. This trial was registered at ClinicalTrials.gov (Available online: https://clinicaltrials.gov/ct2/show/NCT04442620 (accessed on 25 February 2022)). Further information on study protocol can be found elsewhere [18].

### 2.2. Subjects

**Figure 1:**
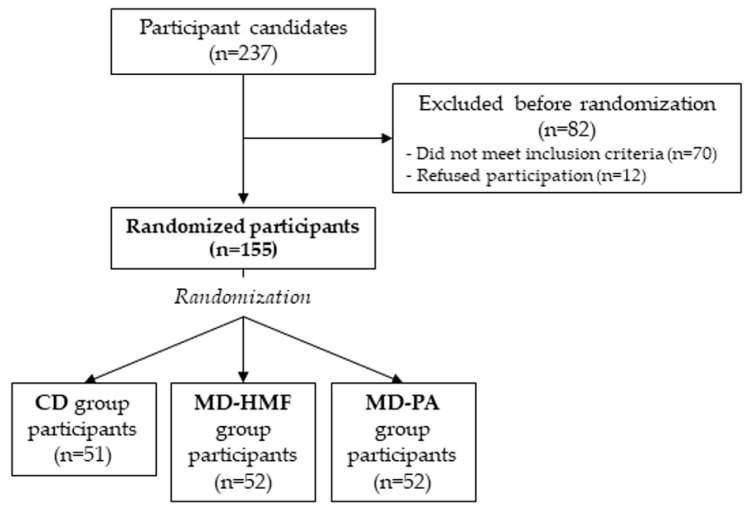
Study flow-chart.

The study population comprised 155 community-dwelling adults (men and women aged 40–60 years), who were overweight or obese (body mass index (BMI) between 27 and 40 kg/m^2^), had a diagnosis by magnetic resonance imaging of NAFLD (Signa Explorer 1.5T, General Electric Healthcare, Chicago, IL, USA) [19], and met at least three criteria for the metabolic syndrome according to the International Diabetes Federation (IDF) [20]. Inclusion and exclusion criteria are available elsewhere [18]. Recruitment and randomization are shown in the study flow-chart (Figure 1). The study protocol and procedures were performed following the Declaration of Helsinki ethical standards and approved by the Ethics Committee of Research of Balearic Islands (ref. IB 2251/14 PI; approved on 26 February 2020). All participants were informed about the study, and they provided written informed consent prior to participation.

### 2.3. Intervention Groups

Selected individuals were randomized into one of three intervention groups, in a 1:1:1 ratio. Randomization was stratified by gender (men/women), type 2 diabetes mellitus (yes/no) and steatosis, using open-source MinimPy desktop minimization program [21], to one of the three intervention groups for 6 months: Conventional diet (CD), AASLD recommendations; MedDiet-high meal frequency (MD-HMF); and MedDiet-physical activity (MD-PA). Figure 1 shows the recruitment process and randomization of participants.

(1) The CD group was based on the American Association for the Study of Liver Diseases (AASLD) recommendations [1]. Patients put into this group were informed to reduce their caloric intake by 25–30% with the following macronutrients distribution: 30% fat, 15% proteins, 55% carbohydrates. They were also told to maintain 25 g/day fiber and <250 mg/day cholesterol intake. Moreover, it was recommended that they walk 10,000 steps per day minimum.

(2) The MD-HMF group was based on the RESMENA diet [22], with 7 meals a day of Mediterranean diet and the highest calorie intake early in the day. Patients were asked to reduce their caloric intake by 25–30% with the following distribution: 30–35% fat, 25% proteins, 40–45% carbohydrates. It was recommended that they walk at least 10,000 steps per day.

(3) The MD-PA group followed the Mediterranean diet recommendations [23] with a meal frequency of 4 or 5 meals a day. Patients put into this group were informed to reduce their caloric intake by 25–30% with the following macronutrients distribution: 35–40% fat, 20% proteins, 40–45% carbohydrates. In terms of physical activity, it was recommended that they did 35-min interval training sessions three times per week: 2 days instructor-led in on-site training sessions, and 1 day of a remote-prescribed training session. The 35-min sessions consisted of a 5-min warm-up, 20-min interval training and 10-min breathing and stretching. The 20-min interval training included 5 tests of moderate intensity with respective recovery seconds between each test. These tests were changed weekly by trained personnel so as not to be monotonous. The remote-prescribed training session was made available to participants through videos sent by WhatsApp or email. The physical activity was personalized, so the intensity of the tests was adjusted to the physical condition of each participant. However, the weekly intensity of aerobic physical activity was equivalent to 10,000 daily steps in terms of caloric expenditure; that is 400 kcal for an adult person who weighs 70 kg.

### 2.4. Lifestyle Recommendations

The study’s personnel tried to motivate participants throughout the study to change their unhealthy lifestyles. The lifestyle recommendations were not to smoke, not to drink alcohol, to follow the diet prescribed, according to their randomization group, to practice regular PA and, at the same time, to follow the previous recommendations of their primary doctors (if applicable). Recommendations could change depending on the evolution of the participants.

### 2.5. Physical Activity and Functional Fitness Assessment

To assess PA as mean weekly time, at baseline and at the 6-month visit, participants reported their leisure and household activities by means of the Spanish version of the Minnesota Leisure Time Physical Activity Questionnaire. The reported energy expenditure was presented as metabolic equivalent of task (MET) in minutes/week [24,25].

Physical fitness was determined with a set of tests at baseline and at the 6-month visit with the ALPHA-FIT test battery [26], and Chester-step test [27]. The used tests were: (1) One-leg balance to assess postural control. If they did not manage to keep the balance 60 s, they had another evaluation. (2) Standing hand grip to assess static grip muscular strength. Participants had to use a handgrip dynamometer (Takei TKK 5401, Tokyo, Japan, range = 5–100 kg, precision = 0.1 kg) with their dominant arm. They had two evaluations. (3) Sitting hand grip to assess sitting grip muscular strength. The same as the previous one, but in a seated position. (4) Jump-and-reach to assess extensor power of lower extremities. There were two evaluations. (5) Modified push-up to assess endurance capacity of the upper extremities’ muscles and trunk. There was only one evaluation and an instructor counted correct push-ups in 40 s. Further details about these tests can be found elsewhere [26]. (6) Chester-step to assess maximal aerobic capacity. Participants had to step on and off a 15 cm high step (Chester Step Test Single Step 15 cm Height, Cartwright Fitness Limited, Huntington, Chester CH3 6DF, UK) for two minutes at the rhythm of a metronome. Further details about Chester-step can be found elsewhere [27]. Finally, maximum oxygen volume (VO_2_ max.) was calculated with Chester-step Software [28].

Tests were performed in the same order as explained above and on the same day by trained personnel. Instructors took the best mark of each test and results were contrasted with a normal range of scores depending on sex and age [26].

Subjects wore an accelerometer (ActiGraph wGT3X-B; ActiGraph LLC, Pensacola, FL, USA) for 7 days, which recorded PA intensities in weekly minutes (sedentary, light, moderate and vigorous; although this latter does not appear in results because no participant recorded vigorous intensity), sleep efficiency and weekly steps. Energy expenditure was presented as metabolic equivalent of task (MET)·minute/week.

A Functional Fitness Score was created to know the global physical function of the studied subjects so as to provide them with PA alternatives and better health promotion [26]. Tests that formed this Functional Fitness Score were: one-leg balance, standing hand grip, jump-and-reach, and modified push-up. The median, by gender, of these four tests was the cut-off value for the Functional Fitness Score, considered the normal range. A score below the median received 0 points (physical fitness lower than median by gender or normal range) and above the median received 1 point (physical fitness higher than median by gender or normal range). A Functional Fitness Score of 4 was the best.

An Accelerometer Fitness Score was also created equally to that above with sedentary, light, and moderate intensities and sleep efficiency. An Accelerometer Fitness Score of 4 was the best.

### 2.6. Other Health Outcomes

Information related to age, gender, education level, marital status (married; divorced; single; or widow), socioeconomic status (low, medium, high), smoking habit (no, ≥1 cigarette/day) and alcohol consumption (no, <7 drinks/week, ≥7 drinks/week) were obtained.

### 2.7. Statistics

Analyses were performed with the SPSS statistical software package version 27.0 (SPSSS Inc., Chicago, IL, USA). Data are shown as mean, standard deviation (SD). Difference in prevalence among groups was tested using χ^2^ (all p values were two-tailed). Prevalence was expressed in sample size and percentage. Normal assumption for continuous data was assessed with Kolmogorov-Smirnov and Shapiro-Wilk tests (according to sample size). Outcomes showing no normal distribution were log-transformed before analysis. Nevertheless, they were presented as untransformed data in the tables for easier interpretation. Baseline analysis of sociodemographic characteristics were tested with one-way ANOVA and Bonferroni’s post-hoc analysis for continuous variables and χ^2^ for prevalence. A Paired sample t-test was used to compare changes from baseline to 6 months of intervention in physical activity parameters within each of the three intervention groups. Changes in physical activity during the 6 months, according to three intervention groups (between-group), were assessed by the Generalized Linear Model. The effect of the intervention was examined by using repeated-measure ANCOVA with two factors: time (baseline vs 6 month) as repeated measure and group (CD, MD-HMF, MD-PA). The Bonferroni post hoc test was performed to compare the changes of each group within, and between, groups. All *p*-values were two-tailed with *p* < 0.05.

## 3. Results

Table 1 shows baseline sociodemographic characteristics of participants in the three intervention groups.

Changes in the physical activity tests at baseline and 6 months follow-up in the three intervention groups are shown in Table 2. There was a significant increase in terms of standing handgrip in the CD (1.1 kg) and MD-PA (1.3 kg) groups from baseline to 6 months.

Looking at the differences over time between groups, the CD group had a higher improvement standing handgrip (1.1 kg) than the MD-HMF group, which decreased in standing handgrip (−0.2 kg). As for endurance of the trunk and upper extremities measured by modified push-ups, only the MD-HMF (1.8 reps) and MD-PA (0.8 reps) groups showed improvement. At 6 months, MD-PA group participants could make more modified push-up repetitions (0.8 reps) than the CD group (0.1 reps).

The fitness score test was negative in all groups; so, participants in the CD group (−0.6), MD-HMF (−0.4) and MD-PA (−0.8) decreased their overall score obtained at 6 months in balance, strength and seated manual pressure, jumping and push-ups tests. Between groups, the MD-PA had a slightly worse score than the CD group. The CD and MD-PA groups showed major improvement in sitting handgrip at 6 months, 1.4 kg, and 1.9 kg, respectively. The MD-PA group increased sitting manual strength more than the MD-HMF (−0.4 kg) group, which decreased. The CD group had more sitting manual strength than the MD-HMF group. Similar results were obtained using Chester-step between the groups at 6 months. The MD-HMF group had the highest decrease in aerobic capacity (−1.9 mL O_2_/kg/min) compared to CD (−0.2 mL O_2_/kg/min) and MD-PA (2.7 mL O_2_/kg/min) groups. The MD-HMF group showed worse results at 6 months, and the MD-PA group showed a significant improvement.

Table 3 shows changes in physical activity parameters from accelerometers at baseline and 6 months follow-up in the three intervention groups. Regarding light intensity from accelerometers, the CD group increased minutes per day at 6 months (25.1 min/day), while the MD-PA group decreased these minutes per day (−19.3 min/day). The MD-PA group decreased light intensity per day considerably more than the CD (25.1 min/day) and MD-HMF (7.3 min/day) groups. In terms of moderate intensity, the MD-PA group showed increased minutes per day at 6 months (7.5 min/day). This increase was higher than the CD (−20.5 min/day) and MD-HMF (−9.3 min/day) groups. With reference to BMI, participants in all groups showed decreased BMI at 6 months of study. The MD-HMF group decreased BMI more (−3.0 kg/m^2^) than the CD (−2.2 kg/m^2^) and MD-PA (−2.0 kg/m^2^) groups. Finally, only the CD group reported more METs per day over time (0.1 MET/day) and were higher than the MD-HMF (0.0 MET/day) group.

## 4. Discussion

The current results showed that patients in the CD group had higher standing handgrip strength than patients in the MD-HMF group at 6 months, who decreased their manual strength (−0.2 kg). Both groups had the same PA recommendation: walking 10,000 steps daily. The difference between them is the dietary intervention. Recent literature showed that handgrip strength can prevent NAFLD, as muscular strength is important to predict NAFLD risk [29]. This is why regular PA is recommended, because it improves physical and musculoskeletal fitness [30]. Accordingly, the explanation for current results could be that the MD-HMF group was more focused on diet than PA, since they had to have seven meals a day, which is not normal. In contrast, the CD group had to have the more normal intake of five daily meals, so they were more focused on PA. Similar results were obtained for sitting handgrip, but, in this case, the MD-PA group also had more handgrip strength in a seated position than the MD-HMF group at six months. These results confirm that regular PA improves physical and musculoskeletal fitness [30].

The current results are aligned with those of previous authors, who analyzed the effect of resistance exercises, such as push-ups, on NAFLD [31]. Regular practice of push-ups, among other exercises, affects muscle strength and improves metabolic parameters related to NAFLD [31]. Current participants showed similar results; so, the MD-PA group, that performed regular PA with push-up practice, managed to perform more repetitions at 6 months than the CD group, whose participants only walked for 6 months. These results suggested that improving muscle strength allows more sustained repetitions of push-ups.

In the current study, a fitness score test was introduced as an alternative tool to assess some functional tests in adult participants with NAFLD. At six months, the fitness score test was negative for the CD and MD-PA groups. This means that both groups worsened their overall functional fitness. However, patients in the MD-PA group had a slightly more negative fitness score test than patients in the CD group. Logically, it should be the other way around. That is, a regular PA program should be more effective in improving global functional fitness than only walking, which would support existing literature [32]. The explanation could be that the CD group did more PA in addition to the recommended 10,000 daily steps, while the MD-PA group might not have strictly adhered to the recommended exercises during the remote sessions. For this reason, the fitness score test was useful, because assessing tests separately could result in obtaining different results.

The Chester-step test measures aerobic capacity or VO_2_ max., i.e., general cardiorespiratory fitness or endurance [27]. Regular PA can increase VO_2_ max. by 100% in unfit people, and 20–40% in moderately fit people. Jogging, swimming, or cycling are some of the exercises that improve VO_2_ max [33]. Our current population confirms these references. The MD-PA group significantly increased their VO_2_ max., more so than the MD-HMF group, which showed a decrease in VO_2_ max. at six months. Training sessions three times per week, as was the case for the MD-PA group, increased aerobic capacity more than only walking. At the same time, although both the CD and MD-HMF groups showed decreased aerobic capacity (negative results), patients in the CD group had a minor loss of aerobic capacity compared to patients in the MD-HMF group. The explanation may be the same as given above for manual muscle handgrip strength.

In terms of intensities measured by accelerometers, the MD-PA group decreased light intensity PA more than the CD and MD-HMF groups at 6 months. In fact, the latter two groups increased light intensity PA, in contrast to the MD-PA group. The same results were obtained but in reverse with moderate intensity. The MD-PA group increased moderate intensity more than the CD and MD-HMF groups, while these latter decreased moderate intensity at 6 months. A trial, which also measured PA intensities with accelerometers, reported that light intensity of PA has health benefits. It can be positive to cardiometabolic diseases and can reduce mortality risk. This trial suggests that PA recommendations should advocate at least moderate desired physical intensity, but, for inactive people, the inclusion of light PA is highly recommended [34]. This evidence is in line with current results. The CD and MD-HMF groups increased light intensity because their participants were only informed to walk. Nevertheless, the MD-PA group were informed to practice 35-min interval training sessions three times per week, and the intensity of the exercises was moderate.

We found that subjects in the CD group reported more MET per day at 6 months than subjects in the MD-HMF (who did not report any change). Reported MET figures came from the Minnesota Leisure-time Physical Activity Questionnaire, an interview about different physical activities for 12 months. There are a total of 63 activities, including garden and home activities too. The frequency of each activity is the average number of times per month, and it has an intensity code, which, together with the duration of the exercise, is expressed as MET·min·day^−1^ [35]. In our case, the CD and MD-HMF groups did the questionnaire at the baseline and at six months, so only six new months were included and not a whole year. The PA recommended was the same for both groups, but at the six-month intervention, the CD group reported more MET per day. Maybe participants in this group did more activities than recommended, whereas the MD-HMF group did not change their PA routine. However, it should be kept in mind that reported METs are a more subjective measure than METs measured by an accelerometer, so it is advisable to compare the results [36]. In the current trial, no significant changes in the METs measured by accelerometer were observed.

### Strengths and Limitations

Scientific evidence tackling the effect of diet and PA on NAFLD is limited. The first strength of the present study is that it contributes to increasing knowledge about changes in PA parameters between three different dietary and PA intervention groups at six months in adults with NAFLD and MetS. Secondly, it includes a large proposal of PA parameters, different measurement methods, and a whole vision of functional tests using the Fitness Score Test as an alternative tool. Other strengths of the present study include the three nutritional interventions. The longitudinal design provides more evidence than cross-sectional designs. The standardized protocol followed reduces the risk of information bias. On top of strengths, our findings could be easily implemented into clinical practice. This strength is very important because there is no existing alternative treatment for NAFLD yet [37]. Nonetheless, the present study has some limitations. The main limitation would be the small sample size. Other limitations are the subjectivity of the Minnesota questionnaire, even if validated. The different physical condition of the subjects, which prevented everyone performing all the tests correctly, is another limiting factor. Lastly, people in the FLIPAN trial were between 40–60 years old, and this is a limitation when it comes to extrapolating results to all the population.

## 5. Conclusions

A lifestyle six-month intervention with diet and regular PA improved functional fitness in middle-aged patients with NAFLD and MetS. Aerobic capacity improved in patients who followed a Mediterranean diet and regular training sessions for 6 months.

## Figures and Tables

**Table 1:** Baseline sociodemographic characteristics of the studied subjects.

	CD (*n* = 51)	MD-HMF (*n* = 52)	MD-PA (*n* = 52)		
	Mean (SD)	Mean (SD)	Mean (SD)		*p*
Age (years) ^#^	53.2 (8.6)	51.9 (7.6)	52.1 (6.1)		0.295
Education (years) ^#^	16.3 (6.6)	15.4 (4.9)	17.0 (7.4)		0.034
	*n* (%)	*n* (%)	*n* (%)		
Gender					0.586
Male	30 (58.8)	33 (63.5)	31 (59.6)		
Female	21 (41.2)	19 (36.5)	21 (40.4)		
Marital status					0.404
Single	5 (9.8)	4 (7.7)	5 (9.6)		
Married	39 (76.5)	40 (76.9)	38 (73.1)		
Divorced	6 (11.8)	8 (15.4)	8 (15.4)		
Widow	1 (2.0)	0 (0.0)	1 (1.9)		
Socioeconomic status					0.097
Low	16 (64.0)	20 (76.9)	19 (73.1)		
Medium	8 (32.0)	5 (19.2)	5 (19.2)		
High	1 (4.0)	1 (3.8)	2 (7.7)		
Smoking habit					0.078
No	43 (87.8)	39 (81.2)	46 (88.5)		
≥1 cigarette/day	6 (12.2)	9 (18.8)	6 (11.5)		
Alcohol consumption					<0.001
No	20 (39.2)	12 (23.1)	10 (19.2)		
Yes, <7 drinks/week	23 (45.1)	32 (61.5)	32 (61.5)		
≥7 drinks/week	8 (15.7)	8 (15.4)	10 (19.2)		

Abbreviations: CD: Conventional diet; MD-HMF: Mediterranean diet-high meal frequency; MD-PA: Mediterranean diet-physical activity. ^#^ log-transformed. Difference in means between groups were tested by ANOVA test. Differences in prevalence’s across groups were assessed using χ^2^.

**Table 2:** Changes in physical activity tests within, and between, intervention groups at 6 months versus baseline.

		CD (*n* = 41)	MD-HMF (*n* = 44)	MD-PA (*n* = 47)	
		Mean (SD)	Mean (SD)	Mean (SD)	Time * Group
Motor fitness tests					
One-leg balance (s)	Baseline	42.7 (21.6)	39.4 (20.2)	43.0 (22.2)	0.570
	6 months	48.3 (18.1)	41.6 (20.8)	49.6 (17.0)	
	Δ	5.6 (15.0) *	2.2 (16.4)	6.5 (19.0) *	
Standing hand grip (kg)	Baseline	37.7 (15.2)	38.8 (11.9)	37.2 (10.9)	0.049
	6 months	38.7 (14.1)	38.6 (11.9)	38.5 (13.3)	
	Δ	1.1 (3.6) *^,a^	−0.2 (6.0) ^a^	1.3 (5.7) *	
Jump-and-reach (cm)	Baseline	22.9 (8.5)	21.5 (8.5)	26.6 (12.7)	0.052
	6 months	23.0 (10.1)	21.5 (9.1)	24.9 (8.4)	
	Δ	0.1 (9.6)	0.0 (6.5)	−1.7 (9.4)	
Modified push-up (reps)	Baseline	7.9 (5.6)	6.8 (4.2)	7.2 (3.3)	0.008
	6 months	8.0 (4.6)	8.6 (4.2)	8.0 (4.6)	
	Δ	0.1 (4.3) ^b^	1.8 (4.0) *	0.8 (3.6) *^,b^	
Fitness score tests	Baseline	1.9 (1.4)	1.9 (1.3)	2.3 (1.5)	0.029
	6 months	1.3 (1.2)	1.5 (0.9)	1.5 (1.1)	
	Δ	−0.6 (0.9) *^,b^	−0.4 (1.0) *	−0.8 (0.9) *^,b^	
Sitting hand grip (kg)	Baseline	36.0 (14.2)	39.5 (11.4)	35.9 (12.4)	<0.001
	6 months	37.4 (14.0)	39.1 (12.4)	37.8 (13.0)	
	Δ	1.4 (4.5) *^,a^	−0.4 (5.7) ^a,c^	1.9 (5.3) *^,c^	
Chester-step (mL O_2_/kg/min)	Baseline	32.5 (8.3)	35.2 (9.2)	35.8 (7.0)	<0.001
	6 months	32.2 (9.8)	33.2 (7.2)	38.5 (7.8)	
	Δ	−0.2 (9.1) ^a^	−1.9 (5.4) *^,a,c^	2.7 (8.3) *^,c^	

Abbreviations: Δ: Change between baseline and 6 months; CD: Conventional diet; cm: centimeters; kg: kilograms; MD-HMF: Mediterranean diet-high meal frequency; MD-PA: Mediterranean diet-physical activity; min: minutes; O_2_: oxygen; reps: repetitions, s: seconds. Data analyzed by two-way repeated measures ANCOVA. Different letters indicate significant differences (*p* < 0.05) between time (*), and between time * group interaction (a, b, c) by the Bonferroni post-hoc test (*p* < 0.05). All variables were log-transformed.

**Table 3:** Changes in physical activity parameters from accelerometers within and between intervention groups at 6 months versus baseline.

		CD (*n* = 41)	MD-HMF (*n* = 44)	MD-PA (*n* = 47)	
		Mean (SD)	Mean (SD)	Mean (SD)	Time * Group
Intensity PA (accelerometer)					
Sedentary (min/day)	Baseline	641.8 (116.8)	639.7 (91.7)	803.5 (191.8)	0.301
	6 months	629.4 (116.0)	648.4 (114.5)	803.3 (195.9)	
	Δ	−12.4 (89.0)	8.7 (88.9)	−0.3 (73.4)	
Light (min/day)	Baseline	508.5 (106.3)	512.4 (83.2)	478.9 (114.9)	<0.001
	6 months	533.6 (93.8)	519.6 (85.7)	459.6 (116.5)	
	Δ	25.1 (64.2) *^,b^	7.3 (47.6)^c^	−19.3 (55.2) *^,b,c^	
Moderate (min/day)	Baseline	242.5 (122.1)	198.5 (71.7)	90.1 (113.2)	0.004
	6 months	221.9 (94.8)	189.2 (47.8)	97.5 (118.0)	
	Δ	−20.5 (88.3) ^b^	−9.3 (73.9) ^c^	7.5 (42.9) *^,b,c^	
Sleep efficiency (%)	Baseline	91.2 (4.2)	92.5 (3.4)	92.3 (3.5)	0.781
	6 months	92.0 (3.1)	92.9 (2.2)	92.7 (3.0)	
	Δ	0.7 (3.0) *	0.5 (2.1)	0.4 (3.8)	
Accelerometer fitness score	Baseline	2.1 (0.6)	2.1 (0.7)	1.9 (0.8)	0.855
	6 months	2.2 (0.8)	2.3 (0.8)	1.9 (0.7)	
	Δ	0.1 (0.8)	0.3 (1.0) *	0.0 (0.7)	
Steps (steps/day)	Baseline	14,789.5 (5262.0)	14,258.5 (2883.8)	12,954.7 (3322.4)	0.462
	6 months	15,267.9 (3649.6)	22,419.4 (32,989.6)	13,305.7 (2874.5)	
	Δ	478.4 (4501.7)	8160.8 (32,273.9)	350.9 (2644.9)	
Energy expenditure					
BMI (kg/m^2^)	Baseline	33.5 (3.5)	34.2 (4.1)	33.7 (3.1)	<0.001
	6 months	31.4 (3.9)	31.2 (3.6)	31.8 (3.4)	
	Δ	−2.2 (2.4) *^,a^	−3.0 (2.9) *^,a,c^	−2.0 (2.0) *^,c^	
Measured accelerometer (MET/day)	Baseline	2.0 (0.3)	1.9 (0.3)	2.0 (0.3)	0.160
	6 months	2.0 (0.3)	1.8 (0.2)	2.3 (1.9)	
	Δ	0.0 (0.3)	0.0 (0.3)	0.3 (1.9)	
Reported Minnesota (MET/day)	Baseline	0.4 (0.3)	0.5 (0.4)	0.5 (0.6)	0.024
	6 months	0.5 (0.4)	0.5 (0.3)	0.5 (0.5)	
	Δ	0.1 (0.4) *^,a^	0.0 (0.4) ^a^	0.0 (0.4)	
Measured-Reported (MET/day)	Baseline	1.6 (0.5)	1.4 (0.6)	1.5 (0.6)	0.492
	6 months	1.4 (0.4)	1.3 (0.4)	1.7 (2.2)	
	Δ	−0.2 (0.5) *	−0.1 (0.7)	0.3 (2.2)	

Abbreviations: Δ: Change between baseline and 6 months; CD: Conventional diet; BMI: body mass index; kg: kilograms; m: meters; MD-HMF: Mediterranean diet-high meal frequency; MD-PA: Mediterranean diet-physical activity; MET: metabolic equivalents of task; min: minutes; PA: physical activity. Data analyzed by two-way repeated measures ANCOVA. Different letters indicate significant differences (*p* < 0.05) between time (*), and between time * group interaction (a, b, c) by the Bonferroni post-hoc test (*p* < 0.05). All variables were log-transformed.

## Data Availability

There are restrictions on the availability of data for this trial, due to the signed consent agreements around data sharing, which only allow access to external researchers for studies following the project purposes. Requestors wishing to access the trial data used in this study can make a request to pep.tur@uib.es.

## Clinical Trials Registration

The trial was registered at ClinicalTrials.gov with registry number NCT04442620 (https://clinicaltrials.gov/ct2/show/NCT04442620; accessed on 30 April 2021).

## Abbreviations

AASLD: American Association for the Study of Liver Diseases; BMI: Body mass index; CD: conventional diet; HDL-c: high density lipoprotein cholesterol; IDF: International Diabetes Federation; MD-HMF: Mediterranean diet-high meal frequency; MD-PA: Mediterranean diet-physical activity; MET: metabolic equivalent of task; MetS: metabolic syndrome; NAFLD: non-alcoholic fatty liver disease; NASH: non-alcoholic steatohepatitis; SD: standard deviations; PA: Physical activity; VO_2_ max.: maximum oxygen volume.
